# The China Space Station: a new opportunity for space science

**DOI:** 10.1093/nsr/nwab219

**Published:** 2021-12-04

**Authors:** Yidong Gu

**Affiliations:** Technology and Engineering Center for Space Utilization, Chinese Academy of Sciences, China

The space station is a large-scale and comprehensive research facility capable of long-term human habitation in near-Earth space, with systematic support by space transport and widely covered tracking, telemetry, command and communication. It allows extensive and long-term research under the special conditions of space and responds to urgent scientific needs in space in a timely manner.

Since the first manned space flight in 1961, two emerging trends have influenced international human space activities. The first trend is manned space exploration represented by the remarkable Apollo program that landed astronauts on the moon. To date, some human space projects have ambitious plans to explore the moon and beyond. The second trend is space science research performed by astronauts in space. Dozens of space laboratories and space stations have been launched, such as the Salute series laboratory, Skylab, the shuttle-borne series laboratory, Mir Space Station and International Space Station currently still in operation. About 20 000 experiments have been conducted, including large-scale scientific research and important technology verification, opening new research directions for space life science and human science, as well as microgravity science that uses microgravity conditions to study the movement of matter and basic physical laws. These experiments also expanded research into space astronomy, solar system science, Earth science, the space environment and new technology, thus playing an irreplaceable role in the human understanding of nature, and promoting scientific and technological advances.

In the past, the development of space science in China was limited by the small scale of space activities and the lack of opportunities for space experiments. Now, construction on the China Space Station (CSS) has begun, with the core module launched in 2021. It will become the most important near-Earth space laboratory for China and the world in the next 10 years. As a result of China's 30-year effort in manned projects, the CSS offers the opportunity for large-scale scientific research that will benefit Chinese scientists and their international peers.

Research in four areas has been planned for the CSS. **Space life science and human research** will investigate the nature of life phenomena and their reactions to microgravity conditions, biomedical issues relating to long-term space exploration, and biotechnology for maintaining human health. **Microgravity physics** will focus on fundamental physical laws, as well as basic research for application in fluid mechanics, thermophysics and combustion and materials science, with a goal of supporting ground industries. **Space astronomy and Earth science** will aim to deepen our understanding of dark matter, dark energy, compact objects, galaxy formation and evolution, and the global changes of the Earth. **New space technology** will develop and verify a new generation of space technology for science and space application.

In order to support research in various fields, the CSS is developing major supporting research facilities such as the 2-meter-diameter China Sky Survey Telescope and the High Energy Cosmic-Radiation Detector. Fourteen experimental racks and three extravehicular exposure devices have been deployed, including the Life and Ecology Research Rack, Biotechnology Research Rack, Fluid Physics Research Rack, Cold Atom Physics Research Rack, High Precision Time-Frequency System, High-Temperature Materials Research Rack, Container-Free Materials Processing Rack and Combustion Sciences Research Rack. Further scientific planning and upgrading of experimental devices will continue in the future.

The CSS is fully open to international researchers, and guides for the open solicitation of research proposals will be regularly issued. International scientists may submit proposals through the China Manned Space Engineering Office, the United Nations Committee on the Peaceful Uses of Outer Space, or national space agencies under bilateral cooperation agreements.

Chinese scientists look forward to extensive cooperation with scientists around the globe, and hope that research activities in the CSS will make a unique contribution to our collective understanding of nature. Through effective dissemination of scientific findings, CSS research programs will promote an understanding and passion for science to the general public, especially to young people.

